# VIP and endothelin receptor antagonist: An effective combination against experimental pulmonary arterial hypertension

**DOI:** 10.1186/1465-9921-12-141

**Published:** 2011-10-26

**Authors:** Sayyed A Hamidi, Richard Z Lin, Anthony M Szema, Sergey Lyubsky, Ya Ping Jiang, Sami I Said

**Affiliations:** 1 Department of Medicine, State University of New York at Stony Brook, NY, USA; 2 Department of Pathology, State University of New York at Stony Brook, NY, USA; 3 Department of Veterans Affairs Medical Center, Northport, NY, USA

**Keywords:** Pulmonary Hypertension, Vasoactive Intestinal Peptide, Endothelin Receptor Antagonist, Pulmonary Vascular Remodeling, Monocrotaline

## Abstract

**Background:**

Pulmonary Arterial Hypertension (PAH) remains a therapeutic challenge, and the search continues for more effective drugs and drug combinations. We recently reported that deletion of the vasoactive intestinal peptide (VIP) gene caused the spontaneous expression of a PH phenotype that was fully corrected by VIP. The objectives of this investigation were to answer the questions: 1) Can VIP protect against PH in other experimental models? and 2) Does combining VIP with an endothelin (ET) receptor antagonist bosentan enhance its efficacy?

**Methods:**

Within 3 weeks of a single injection of monocrotaline (MCT, *s.c*.) in Sprague Dawley rats, PAH developed, manifested by pulmonary vascular remodeling, lung inflammation, RV hypertrophy, and death within the next 2 weeks. MCT-injected animals were either untreated, treated with bosentan (*p.o*.) alone, with VIP (*i.p*.) alone, or with both together. We selected this particular combination upon finding that VIP down-regulates endothelin receptor expression which is further suppressed by bosentan. Therapeutic outcomes were compared as to hemodynamics, pulmonary vascular pathology, and survival.

**Results:**

Treatment with VIP, every other day for 3 weeks, begun on the same day as MCT, almost totally prevented PAH pathology, and eliminated mortality for 45 days. Begun 3 weeks after MCT, however, VIP only partially reversed PAH pathology, though more effectively than bosentan. Combined therapy with both drugs fully reversed the pathology, while preventing mortality for at least 45 days.

**Conclusions:**

1) VIP completely prevented and significantly reversed MCT-induced PAH; 2) VIP was more effective than bosentan, probably because it targets a wider range of pro-remodeling pathways; and 3) combination therapy with VIP plus bosentan was more effective than either drug alone, probably because both drugs synergistically suppressed ET-ET receptor pathway.

## Background

The pathogenesis of pulmonary arterial hypertension (PAH) is incompletely understood, and its treatment remains imperfect. The principal pathologic lesions of pulmonary vascular remodeling, inflammation, and right ventricular hypertrophy (RVH), involve genetic and environmental factors, and are mediated by imbalances in key pathways that either promote or modulate their development [1-3].

Research into the causation and treatment of PAH is heavily dependent on experimental models that mimic the disease. Among these is the uniformly fatal model of PAH induced in rats by monocrotaline (MCT), the toxin derived from *Crotolaria spectabilis *[4]. Despite its imperfections [5,6], this model has long been used to clarify the pathogenesis of PAH, and test the therapeutic potential of new drugs for its management. In the continuing search for more effective therapies, a recent review discussed some of these drugs: Tyrosine kinase inhibitors (platelet-derived growth factor and epidermal growth factor receptor inhibitors), multikinase inhibitors (tyrosine kinase and serine/threonine kinase), elastase inhibitors, Rho kinase inhibitors, endothelial nitric oxide synthase-related agents, survivin inhibitors, HMG-COA reductase inhibitors, and peptides, including vasoactive intestinal peptide (VIP) and adrenomedullin [7].

VIP relaxes pulmonary vascular smooth muscle [8,9], neutralizes a variety of pulmonary vasoconstrictors, including endothelin [10] and hypoxia [11], inhibits airway and pulmonary vascular smooth muscle cell proliferation [12,13], and has broad anti-inflammatory actions [14]. We recently reported that mice with targeted deletion of the VIP gene express a spontaneous phenotype of PAH, without the added stimulus of hypoxia [15]. Treatment of the VIP-deficient mice with VIP corrected the key features of the disease: vascular remodeling, RVH, and lung inflammation [15-17]. VIP has also already been the subject of 2 clinical trials in human PAH, with conflicting results [13,18].

The primary objectives of the present study were to answer 2 questions: a) Is VIP effective in preventing and reversing a model of PAH, such as that induced by MCT, which is both widely used and not directly attributable to VIP deficiency? b) Can the effectiveness of VIP against experimental PAH be enhanced by combining it with another anti-proliferative agent such as bosentan, itself with proven benefit in the MCT model [19]; as well as in clinical PAH [2]? The selection of bosentan seemed particularly appropriate because we had earlier reported that VIP modulated hypoxic pulmonary hypertension in Fawn-hooded rats, by suppressing the expression of both endothelin (ET) and its receptors [11]. Thus, we reasoned that the combination of VIP and bosentan might exert a more powerful effect against the endothelin system, and possibly against other pathways that mediate vascular remodeling [20]. The results validate this expectation.

## Methods

### 

#### Ethics Statement

All experiments and animal care procedures were approved by the Institutional Animal Care and Use Committee (IACUC) at SUNY Stony Brook (Permit number: 20101678) and at Northport VA Medical Center (Permit number: 00328), and were carried out in strict accordance with the recommendations in the Guide for the Care and Use of Laboratory Animals of the National Institutes of Health.

#### Animals

Sprague Dawley rats, 200-230 g, 6-8-week old, were from Taconic Labs (Germantown, NY). All surgical procedures were performed under anesthesia, and every effort was made to minimize suffering.

#### Chemicals and reagents

MCT was from Sigma-Aldrich (St. Louis, MO); VIP was from Bachem Americas Inc. (Torrance, CA); and bosentan was provided by Actelion Pharmaceutical Ltd. (Allschwil, Switzerland).

### Study Design

The study was designed to test 3 objectives:

#### 1) Prevention by VIP

2 groups of rats were injected with MCT (a single *s.c*. injection of 60 mg/kg). Group 1 (*n *= 10) rats received no additional treatment; group 2 (*n *= 10) rats received VIP at 500 μg/kg, *i.p*. (in 0.2 ml PBS), every other day for 3 weeks, beginning the same day as MCT, for a total of 10 injections. This is the same VIP dose regimen we used to reverse the PAH phenotype caused by deletion of the VIP gene (15). A third group of 10 rats, serving as controls, received neither MCT nor VIP. Survival was monitored for 45 days in a fourth group of 8 rats that received a single dose of MCT plus VIP, as above.

#### 2) Reversal by VIP or bosentan

A group of 10 rats received VIP at 500 μg/kg, *i.p*. (in 0.2 ml PBS), every other day for 3 weeks, beginning 3 weeks after the injection of MCT, *i.e.*, after PAH pathology had already developed. Another group of 10 rats received bosentan at 300 mg/kg/day, as food admix (Product # TD.09065, Harlan Laboratories Inc., WI), for 3 weeks, beginning 3 weeks after MCT.

#### 3) Reversal by combination therapy

Three weeks after MCT injection, another group of rats (*n *= 10) received both VIP and bosentan, as above, also for 3 weeks.

### Measurements

All rats were subjected to the following measurements, 3 weeks following the single injection of MCT in the prevention studies, and 3 weeks after the pathology had already developed (6 weeks after MCT) in the reversal experiments.

#### Hemodynamic measurements

Rats were anesthetized with *i.p*. ketamine (100 mg/kg) and Fentanyl (0.05 mg/kg). A 1.4 F Mikro-Tip pressure transducer catheter (Millar Instruments Inc., Houston, TX) was inserted through the right jugular vein and advanced to the right ventricle for digital recording of RV (or PA) systolic pressure.

#### Histological examination & morphometric analysis

The animals were euthanized by an injection of 120 mg/kg of pentobarbital, the chest was opened, and the right main-stem bronchus was tied off. For all histological procedures, the upper and middle lobes of the right lung were fully inflated and fixed by intra-tracheal instillation of 2 ml 10% buffered formalin, then embedded in paraffin. Sections (4 μm thick) were stained with hematoxylin and eosin or Masson's Trichrome for general morphologic and morphometric analysis. Measurements were made of 4 separate pulmonary arterioles from each lung, and averaged to 1 set of values. Only arteries near smaller bronchi or terminal bronchioles, 50-80 μm in diameter, were selected for analysis. The *Image J program*, version 1.34r *(*http://rsb.info.nih.gov/ij*/)*, was used for measurement of total vessel area (μm^2^), luminal area (μm^2^), and inner circumference (μm). Medial area (μm^2^) was calculated as the difference between total and luminal areas. Standard medial thickness (μm) was calculated as the ratio of medial area to inner circumference, as described [15,21].

#### Anatomic assessment of RV hypertrophy

The heart was isolated and placed under a dissecting microscope. Attached vessels and both atria were dissected and removed. The RV wall was cut out, blotted, and weighed, then the left ventricular wall and septum (LV+septum) were treated in the same way. The RV/(LV+Septum) weight ratio was calculated as an index of RV hypertrophy.

#### Assessment of lung inflammation

Lung sections were examined by a pathologist who was blinded to the identities of the samples. Inflammation was graded 0, 1, 2, 3, or 4, based on the intensity and extent of perivascular and peribronchiolar inflammatory cell infiltrates.

#### Survival

After a single injection of MCT, all rats were monitored for 45 days. Mortality data were compared among all experimental groups, by the log-rank test, and Kaplan-Meier plots.

#### Statistical analysis

Quantitative data from all groups were analyzed by ANOVA and *Tukeys post-hoc *test for multiple comparisons. A *P *value of < 0.05 was considered significant.

## Results and discussion

### Results

#### MCT-induced PAH (Table 1, Figures 1 &2)

As reported by others [22], a single injection of MCT resulted in full development of pulmonary vascular pathology within 3 weeks. RV systolic pressure was significantly elevated relative to levels in control, untreated rats. Small pulmonary arteries and arterioles had thickened media (20.4 ± 1.1 μM *vs*. 8.5 ± 0.5 μM, *P*< 0.001, *n *= 10), and narrower lumen than control vessels of similar diameter (50-80 μm). The corresponding ratio of medial area to luminal area in MCT-treated rats was significantly elevated, as was the RV/(LV+septum) weight ratio. The lungs showed perivascular and peribronchiolar inflammatory cell infiltrates, predominantly macrophages and lymphocytes. All MCT-treated rats died within 3-5 weeks after MCT injection, with no mortality noted in the control group.

**Table 1:** The PAH Phenotype in MCT-Injected Rats: Near-Full Prevention by Early Treatment with VIP *

Measurements	Control, untreated	MCT +		*P *value (post hoc)		
		
		0	VIP	†	**	††
RV systolic pressure (mm Hg)	24.3 ± 2.4	61.4 ± 4.8	29.9 ± 1.8	< 0.05	< 0.05	NS
Arteriolar medial/luminal area	0.76 ± 0.07	3.9 ± 0.51	1.5 ± 0.2	< 0.05	< 0.05	NS
RV/(LV+septum) weight ratio	0.26 ± 0.01	0.56 ± 0.03	0.35 ± 0.01	< 0.05	< 0.05	NS
Lung inflammation (0-4)	0.20 ± 0.17	3.0 ± 0.26	0.3 ± 0.21	< 0.05	< 0.05	NS

* VIP treatment was begun on the same day as MCT. ANOVA: p < 0.0001

† MCT alone *vs*. control; ** VIP pretreatment *vs*. MCT alone; **†† **VIP pretreatment *vs*. control.

**Figure 1:**
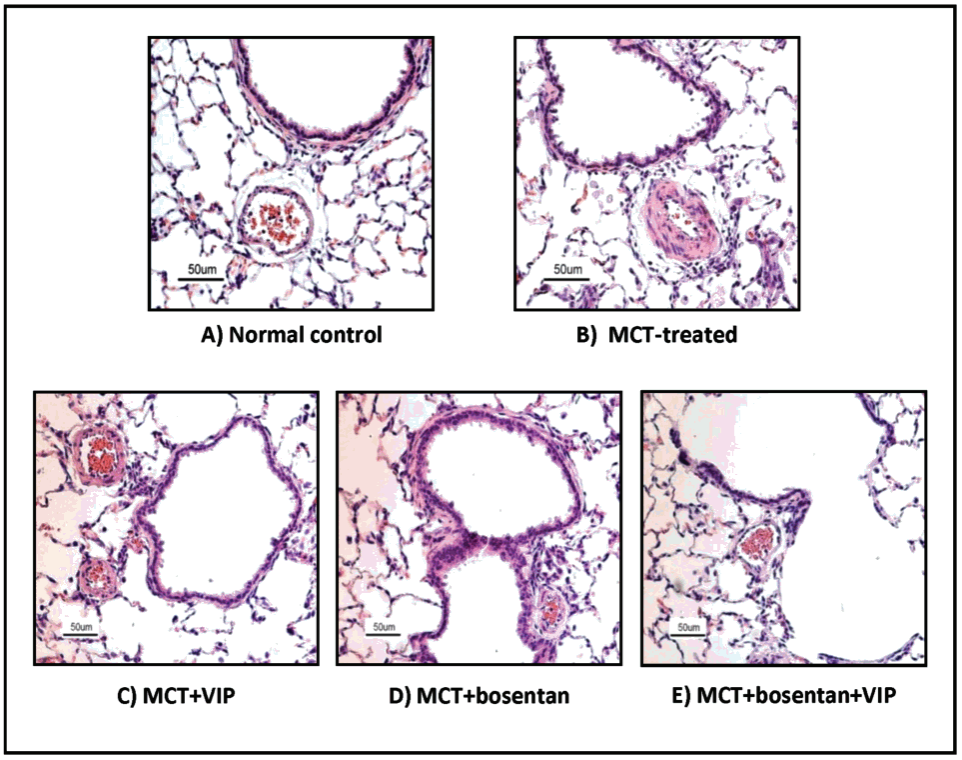
**Rat lung sections (H&E) showing MCT-induced vascular thickening and its attenuation by VIP, bosentan, or their combination**. Small pulmonary arteries from MCT-treated rats had thickened media and narrowed lumen compared to arteries of similar diameter (70-80 μm) from control, untreated rats (A, B, *P*< 0.001). Treatment with either VIP or bosentan (C, D) resulted in significant attenuation of these changes: the ratio of medial area to luminal area in VIP-treated rats was significantly reduced (*P*< 0.05). In rats receiving a combination of VIP and bosentan (E), the medial area/luminal area ratio was almost the same as in control untreated rats (magnification: X20).

**Figure 2:**
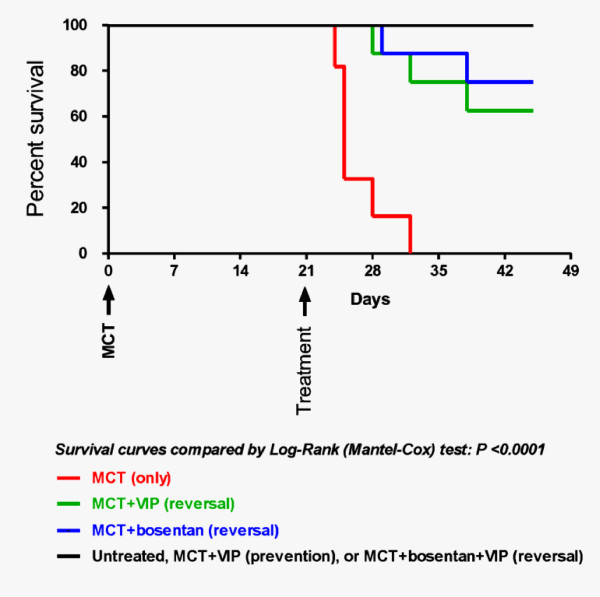
**Kaplan-Meier plot of survival over 45 days**. Rats were observed for 45 days after MCT injection. MCT was uniformly fatal within 35 days. Early (preventive) treatment with VIP was followed by no mortality for 45 days, *i.e*., just as in control, untreated rats. Treatment with either bosentan or VIP alone, begun at 21 days, reduced mortality in MCT-treated rats by 29% (*P*< 0.0001). No mortality was observed during the same time period in rats that received both bosentan and VIP.

#### Prevention of PAH by VIP (Table 1, Figures 1 &2)

In MCT-injected rats that were co-treated with VIP, all hemodynamic, histologic, morphometric, and pathologic findings were statistically unchanged from the control, untreated group. As well, all animals in this group were alive 45 days after the MCT injection.

#### Reversal of PAH by VIP (Table 2, Figures 1 &2)

When VIP treatment was begun 3 weeks after MCT injection, RV systolic pressure was considerably lower than in MCT-treated rats (33 ± 0.5 *vs*. 61.4 ± 4.8 mm Hg, *n *= 5, *P*< 0.05), but still higher than in control, untreated rats (24.3 ± 2.4 mm Hg). The ratio of medial area to luminal area in this group was also well below that in MCT-treated rats (1.68 ± 0.19 *vs*. 3.9 ± 0.51, *n *= 7, *P*< 0.05), but higher than in control, untreated rats (0.76 ± 0.07, *P*< 0.05). The most prominent improvement was in the intensity/extent of inflammatory cell infiltrates, which was down to the minimal value of 0.3 ± 0.21, no different from the control untreated group (0.2 ± 0.17). However, the RV/(LV+septum) weight ratio was only slightly but not significantly reduced (0.51 ± 0.03 *vs*.0.56 ± 0.03, *n *= 10, *P*:NS). Treatment with VIP also markedly reduced mortality (to 29%, *P*< 0.0001).

**Table 2:** Reversal of MCT-Induced PAH by VIP, Bosentan, or Their Combination

Measurements	Control, untreated	MCT +				*P *value (post hoc)		
		
		0	VIP	Bosentan	VIP + Bosentan	*	†	**
RV systolic pressure (mm Hg)	24.3 ± 2.4	61.4 ± 4.8	33 ± 0.5	39 ± 1.2	26.0 ± 1.2	< 0.05	< 0.05	< 0.05
Arteriolar medial/luminal area	0.76 ± 0.07	3.9 ± 0.51	1.68 ± 0.19	1.76 ± 0.19	0.79 ± 0.05	< 0.05	< 0.05	< 0.05
RV/(LV+septum) weight ratio	0.26 ± 0.01	0.56 ± 0.03	0.51 ± 0.03	0.47 ± 0.07	0.34 ± 0.02	*NS*	*NS*	< 0.05
Lung inflammation (0-4)	0.20 ± 0.17	3.0 ± 0.26	0.3 ± 0.21	1.2 ± 0.28	0.3 ± 0.33	< 0.05	< 0.05	< 0.05

Therapy with either drug, or both together, was begun 3 weeks following MCT, i.e., after PH had fully developed. ANOVA: p < 0.0001

* MCT + VIP vs. MCT; † MCT + Bosentan vs. MCT; ** MCT + VIP + bosentan vs. MCT

#### Reversal of PAH by bosentan (Table 2, Figures 1 &2)

Like VIP, bosentan significantly reduced RV systolic pressure, though somewhat less effectively (to 39 ± 1.2 mm Hg). Pulmonary vascular thickening was similarly reduced (medial area/luminal area: to 1.76 ± 0.19), but RV/(LV+septum) weight ratio was only slightly but not significantly reduced, and bosentan was less effective than VIP in attenuating the intensity and extent of inflammatory cell infiltrates (to 1.2 ± 0.3). As with VIP, overall mortality was reduced to 29%, *P*< 0.0001.

#### Reversal of PAH by combination of bosentan and VIP (Table 2, Figures 1 &2)

MCT-injected rats co-treated with VIP and bosentan had RV systolic pressure almost as low as in control, untreated rats (26.0 ± 1.2 *vs*. 24.3 ± 2.4 mm Hg, *n *= 5, *P *= NS), had normal or near-normal medial area/luminal area ratio (*n *= 8, *P *= NS), and almost no inflammatory cell infiltrates. RV/LV+septum ratio was reduced to levels statistically no different from those in control untreated group (0.26 ± 0.01). Finally, no mortality was observed over the 45-day observation period.

### Discussion

Our findings with the induction of PAH following a single injection of monocrotaline in rats are in agreement with reports by other investigators who have studied this experimental model [22]. The pathologic and pathophysiologic features included 4 key elements: a) RV systolic hypertension; b) right ventricular hypertrophy c) perivascular inflammatory cells infiltrates; and d) remodeling of smaller pulmonary arteries, mainly by smooth muscle cell and collagen proliferation. The additional lesion of neointimal proliferation, seen in severe human disease, has been reported in rats receiving MCT following pneumonectomy, or in conjunction with other procedures [5,6].

#### The complex pathogenesis of MCT- induced PAH

Strong evidence supports the view that monocrotaline induces pulmonary vascular pathology via multiple mechanism and pathways, including: activation of: the *endothelin-endothelin receptor *pathway [19]; the *PDGF *(platelet-derived growth factor) pathway, a potent mitogen that stimulates proliferation and migration of pulmonary vascular smooth muscle cells [23]; upregulation of the *serotonin transporter *[24]; increased *serine elastase *activity and proteinase-dependent deposition of tenacin-C, which amplifies the response of smooth muscle cells to growth factors [22]; the *angiotensin *system; and *Rho-kinase *activity [25].

#### A variety of agents attenuate MCT pathology

By the same token, attenuation of the MCT-induced phenotype has been modulated by a variety of agents, including: endothelin receptor antagonists, *e.g. bosentan *[19]; *Imatinib*, an inhibitor of PDGF; M249314, a *serine elastase inhibitor *[22]; *fasudil*, an Rho kinase inhibitor [25]; drugs that increase *cyclic GMP *levels, such as promoters of the *L-arginine-endothelial nitric oxide synthase (eNOS) *pathway [26], and *phosphodiesterase 5 inhibitors*, such as sildenafil [27]; as well as activators of cyclic AMP production, such as *prostacyclin analogs *[28]. In addition, highlighting the important role of inflammation in pathogenesis, specific inhibition of *p38 MAP kinase*, selectively associated with inflammation, attenuated the progression of PAH in MCT-treated rats [29].

#### Critique of the MCT model

1) All features of PAH in the MCT model were significantly more pronounced than in the phenotype we described in mice with deletion of the VIP gene [15]: Thus, RV systolic pressure reached 61.4 ± 5.3 mm Hg *vs*. 29.5 ± 1.1 mm Hg in VIP^-/- ^mice (*P*< 0.001); RV/(LV+Septum) was 0.56 ± 0.03 *vs*. 0.34 ± 0.01 (*P*< 0.001); both vascular remodeling and the inflammatory response were more severe, and survival was considerably more limited in the MCT rat model (5-7 weeks) *vs*. 15 months in VIP^-/- ^mice.

2) The MCT model has been criticized for not expressing intimal proliferation, a feature of human PAH and, overall, for being less of a therapeutic challenge than the human disease [5,6].

3) Many drugs that have successfully reversed MCT lesions in rats have failed to reverse the human PAH lesions. However, reversal of MCT pathology is not always guaranteed, as recently demonstrated for the powerful rapamycin and statin combination [30].

#### A moderate response to bosentan

As predicted from earlier results by other investigators [19], bosentan was moderately effective in reversing all features of PAH in this model, being least effective in correcting RVH. It also reduced mortality over the 45-day observation period, from 100 to 29%.

#### A stronger response to VIP

The anti-PAH effectiveness of VIP is attributable to its wide-ranging modulatory influence on multiple pathways [20]. Analysis of gene expression in lungs of VIP^-/- ^mice revealed that deletion of the VIP gene leads to under-expression of both BMP2 (bone morphogenetic protein II) and BMPR2 [20], the latter being the principal gene mutation predisposing to Idiopathic and Familial PAH [31,32], along with over-expression of key pro-proliferative and pro-inflammatory genes, and under-expression of major anti-proliferative genes [20]. In separate studies, we demonstrated that VIP selectively down-regulates the expression of ET & ET receptors [11]. With such a broad influence on critically important genes controlling pulmonary vascular structure and function, it is not surprising that VIP could have a powerful, though incomplete, effect on the MCT-induced pulmonary pathology. An earlier report described beneficial effects for VIP in MCT-treated rabbits, following cardio-pulmonary bypass [33].

#### Prevention vs. reversal by VIP

Tested for its ability to reverse PAH after its development, VIP treatment resulted in marked reduction in RV systolic pressure, and medial area/luminal area, but only slight reduction in RV hypertrophy. The failure of VIP to induce more pronounced reversal of RVH compared to RV hypertension can be explained by the relative ease of reversing sustained pulmonary vasoconstriction compared to attenuation of proliferative changes in pulmonary vessels and RV. At the same time, mortality was significantly reduced from 100 to 29% (*P*< 0.0001). By comparison, early-treatment with VIP decreased all pathologic features of PAH, including RV hypertrophy, to a greater extent, and ensured full survival of rats injected with an otherwise uniformly fatal dose of MCT. Both in the prevention and reversal studies, VIP practically eliminated lung inflammation. Overall, VIP was fairly effective in this model despite the greater severity of the pathologic lesions, compared to VIP^-/- ^model. The reason VIP treatment was not as fully effective as in the latter model was probably because of the complex underlying genotypic alterations [34].

#### The superiority of the VIP + bosentan combination

The most effective treatment regimen in these studies was the combination of VIP and bosentan. All expressions of PAH: RV systolic pressure, vascular remodeling, RV hypertrophy, and Inflammation, were statistically back to control values. In addition, combination therapy totally eliminated mortality, just as with preventive VIP treatment. The superiority of this combined therapy is probably related to several factors: 1) the wide range of pathways targeted by VIP [20]; 2) the complementary synergistic anti-ET effect resulting from VIP-induced down-regulation of both ET and ET receptor expression, combined with ET receptor inhibition by bosentan [11]; and 3) the potent anti-inflammatory activity of VIP, which assumes special significance in this model in view of the prominent inflammatory response elicited by MCT [35]. Analysis of the influence of this combined therapy on the expression of PAH-related genes and pathways would provide helpful information in this regard.

#### Gene expression alterations

As we reported elsewhere [34], genotypic analysis showed 2 distinct sets of alterations in MCT-treated rats: a) one, similar to that in VIP^-/- ^mice, *i.e.*, alterations that tend to *promote *vascular remodeling and inflammation (*e.g.*, up-regulation of myosin polypeptides, procollagen, and some inflammatory genes); and b) another set of alterations that suggest an effort to *modulate *the PAH. (*e.g.*, up-regulation of *Vip *and *Nos3*). Subsequent treatment with VIP was partially successful in reversing these genotypic abnormalities.

## Conclusions

1) The findings demonstrate that VIP is effective, not only against the PAH phenotype resulting from deletion of the VIP gene, but also effective in preventing, and at least partially reversing, the monocrotaline model of PAH.

2) The combination of VIP with bosentan has special therapeutic advantages, because of the targeting of multiple pathogenetic pathways by both agents: VIP, with its potent, pulmonary vascular-relaxant and anti-proliferative effects, and bosentan with its synergism on the endothelin-endothelin receptor system.

3) Overall, the results are particularly topical, because of the present uncertainty about the therapeutic potential of VIP in human PAH: One clinical trial [13], showed dramatic benefit, while a more recent trial, so far published only as an abstract [18], reported negative results. This report could help introduce a new line of therapy with this peptide, alone or in combination with other drugs, in this devastating disease.

4) While the conclusions of this study may not necessarily apply to human PAH, they give added incentive to the search for appropriate, especially advantageous drug combinations against PAH. In particular, the findings support the emerging hypothesis that, both experimental models and clinical PAH are likely to benefit most from appropriate combinations of anti-proliferative drugs. Ideally, such combinations should be based on genotypic information of the different forms of PAH, in accordance with the concept of pharmacogenomics, which has revolutionized therapy in oncology [36].

## List of Abbreviations

***BMP2: ***Bone Morphogenetic Protein II; ***eNOS: ***Endothelial Nitric Oxide Synthase; ***MCT: ***Monocrotaline; ***PAH: ***Pulmonary Arterial Hypertension; ***PH: ***Pulmonary Hypertensio; ***PDGF: ***Platelet-Derived Growth Factor; ***PVR: ***Pulmonary Vascular Remodeling; ***RVH: ***Right Ventricular Hypertrophy; and ***VIP: ***Vasoactive Intestinal Peptide.

## Competing interests

The authors declare that they have no competing interests.

## Authors' contributions

**SAH: **Design of the study, animal experiments, hemodynamic studies, morphometric analysis, data acquisition and statistical analysis, and drafting the manuscript; **RZL: **Hemodynamic studies and data acquisition; **AMS: **Animal experiments, with reference to inflammatory and immunologic responses and hemodynamic studies; **SL: **Supervised and evaluated pathologic and histological examination of the lungs; **YPJ: **Hemodynamic studies data acquisition; **SIS: **Design and conduct of the study, drafting and revising the manuscript for important intellectual content and final approval of the version to be published. All authors have read and approved the final manuscript.
